# Low Dispersity and High Conductivity Poly(4-styrenesulfonic acid) Membranes Obtained by Inexpensive Free Radical Polymerization of Sodium 4-styrenesulfonate

**DOI:** 10.3390/membranes8030058

**Published:** 2018-08-07

**Authors:** Victor Raul Sepulveda, Ligia Sierra, Betty Lucy López

**Affiliations:** Grupo de Investigación Ciencia de los Materiales, Instituto de Química, Facultad de Ciencias Exactas y Naturales, Universidad de Antioquia, Calle 70 No. 52-21, Medellín 050010, Colombia; ligia.sierra@gmail.com (L.S.); bettylope@gmail.com (B.L.L.)

**Keywords:** low dispersity, free radical polymerization, sodium 4-styrenesulfonate

## Abstract

Controlled polymerizations are often used to synthesize polymers with low dispersity, which involves expensive initiators, constrained atmospheres, and multi-step purifying processes, especially with water soluble monomers. These drawbacks make the synthesis very expensive and of little industrial value. In this report, an inexpensive free radical polymerization of sodium 4-styrenesulfonate, using benzoyl peroxide as initiator in water/*N*,*N*-dimethylformamide solutions, is presented. After polymerization, an easy fiber precipitation method is implemented to extract and purify the polymer, obtaining conversions up to 99%, recoveries up to 98%, and molecular weight dispersities in the range of 1.15–1.85, where the pseudo-controlled behavior is attributed to a thermodynamic limiting molecular weight solubility. Three different methods were used to bring the polymer to its acid form, obtaining Ion Exchange Capacities as high as 4.8 meq/g. Finally, polymeric membranes were prepared and reached conductivities up to 164 mS/cm, which makes them good candidates as proton exchange membranes in fuel cells.

## 1. Introduction

Water soluble homopolymers and copolymers of sulfonated polystyrenes [1] are of great interest in the renewable energy field, being used as hole transporters in organic solar cells [2,3], ionic exchangers in polymer-lithium batteries [4,5], and proton conductors in fuel cells [6,7,8]. Since it is very important to obtain the polymers with narrow molecular weight dispersity, in order to control their properties, many authors have proposed different approaches to reach this aim. As a first approach, a sulfonation process can be performed on pre-synthesized nearly monodisperse polystyrene [9,10], but the reaction is far from 100% efficient, suffers from high crosslinking, uses very aggressive reagents (as concentrated sulfuric or chlorosulfonic acids), and requires controlled atmospheres [11]. These drawbacks and the fact that styrenesulfonic membranes can be degraded in the aggressive operating conditions of fuel cells [12,13], as the oxyradicals produced in the cathode and the high acid concentrations, resulted in this homopolymer being rapidly abandoned as a proton exchange membrane.

Another approach is using the sulfonated monomer, ensuring a full sulfonation degree and low/no crosslinking. As the monomer is water soluble but insoluble in many solvents, common aqueous controlled polymerization deviates from the ideal living control, due to the interaction of the different species involved in the reaction with the water molecules. Atom Transfer Radical Polymerization (ATRP) [14,15], Nitroxide Mediated Polymerization (NMP) [16,17], and reversible addition fragmentation chain transfer (RAFT) polymerization [1,18] are common controlled polymerizations that have been used to polymerize the sodium 4-styrenesulfonate (NaSS) in aqueous solutions, but the use of expensive initiators, constrained atmospheres, and the multiple steps required to purify the products and the low molecular weight reached, are common drawbacks that limit its use in real world applications.

From a different perspective, one of the most versatile methods to polymerize styrenesulfonic monomers is free radical polymerization using peroxides as initiators in pure *N*,*N*-dimethylformamide [19,20] (DMF), where benzoyl peroxide (BPO) is commonly used for industrial applications because of its low cost and well-known half-life time. In this type of polymerizations, the reaction is not kinetically controlled and polymers can reach high dispersities even at low molecular weights. Furthermore, for the use in proton conductive membranes, it is important to have a clean polymer free of DMF because it deactivates the available sites for proton mobility [21,22].

In this paper, we report an inexpensive free radical polymerization of NaSS in water/DMF solutions, using BPO as initiator, which resulted in a polymerization with high molecular weight and low dispersity, and a methodology to purify and protonate the polymer to be used as ion exchange membranes.

## 2. Experimental

### 2.1. Materials

Sodium 4-styrenesulfonate (99%) was obtained from Alfa Aesar (Tewksbury, MA, USA); *N*,*N*-dimethylformamide (DMF, 99%), sulfuric acid (98%), and hydrochloric acid (36%) were obtained from Sigma-Aldrich (St. Louis, MO, USA); benzoyl peroxide (BPO, 75%, humidified with ~25% of H_2_O) was obtained from Panreac AppliChem (Barcelona, Spain); acetone EMSURE^®^ was obtained from Merck (Kenilworth, NJ, USA); ultrapure type I water was produced with an EMD Millipore Synergy^®^ UV water purification system (Darmstadt, Germany); Spectra/Por type 3 dialysis membrane (3.5 kDa) was obtained from Spectrum Laboratories Inc. (Waltham, MA, USA). All reagents were used without further purification, except for the monomer that was dried in a vacuum oven at 50 °C overnight prior to use.

### 2.2. Polymerization

No atmospheric control was employed during the whole process, but the flask was sealed with a rubber septum and a needle was used to avoid overpressure. In a typical procedure 2 g of dried NaSS monomer is slowly added to 9 mL of a water/DMF mixture in a 20 mL round bottom flask, stirred at 300 rpm and heated to 90 °C in an oil bath for 30 min. Then 53 mg of BPO (initiator to monomer 2 wt.%) is dissolved in 1 mL of DMF (this volume is taken in account in the water/DMF ratio) and added to the system via syringe. A pre-polymerization procedure was carried out at 90 °C for 2 h. The temperature is then raised to 105 °C and further polymerized for 16 h. The water/DMF volume ratio was varied from 1/9 to 9/1 to affect the polymer solubility. After polymerization, a yellowish gel precipitates which is extracted by decantation (PNaSS). Molecular weight measurements were performed in a Viscotek GPCmax VE2001 (Malvern Panalytical Ltd., Roystone, UK) with a TDA305 Triple detector (Malvern Panalytical Ltd., Roystone, UK), using an A6000M column (Malvern Panalytical Ltd., Roystone, UK) at 35 °C, in a mobile phase of 0.1 M NaSO_4_ at 0.5 mL min and a Pullulan Standard Set is used for calibration.

### 2.3. Polymeric Fiber Precipitation and Protonation

In all cases, the extracted polymer gel (about 1.5–4 g depending on the system) is added to 10 mL of water in a 20 mL vial until a translucent viscous solution is reached (PNaSS solution). Then, 80 mL of acetone are placed in a 100 mL flask and stirred at 700 rpm using a cross spin magnetic stir bar (a vortex is formed) and PNaSS solution is added dropwise. The polymer precipitates as a fibrous sponge which is easily extracted with tweezers after each 2.5 mL of added PNaSS solution. The acetone is renewed after 5 mL of added solution (after the second extraction), in order to avoid overhydration. Finally, the solid PNaSS is dried in a vacuum oven at 50 °C for 24 h and weighed. This procedure of polymer solution/fiber precipitation is performed threefold to ensure a clean polymer.

Three methods were used to protonate the polymer. In the first two cases, 2 g of dry polymer is dissolved in 10 mL of 2 M hydrochloric acid (sample PSSH82-HCl) or 2 M sulfuric acid (sample PSSH82-SA) and stirred at 300 rpm for 24 h, followed by fiber precipitation as described above. In the third case, 2 g of polymer is dissolved in 10 mL of water in a 20 mL vial followed by a dropwise addition (~0.5 mL/min) of 3 mL of concentrated sulfuric acid (PSSH82-SAP), under continuous stirring at 300 rpm; to avoid overheating, the vial is chilled in an ice bath. During the process a light brown gel precipitates, which is stirred up to 24 h and extracted by decantation. All samples were subsequently dialyzed using ultrapure water until pH reaches neutrality (up to 12 h) and finally lyophilized to obtain a yellowish solid sponge.

Ion Exchange Capacity was measured by titration method and calculated using Equation (1), where *V_NaOH_, C_NaOH_*, and *W_PDry_* are the volume of NaOH consumed in the titration (mL), the concentration of NaOH (mmol/mL) and the corrected weight of the sample (g), respectively. In a typical procedure ~200 mg of polymer is dissolved in 40 mL of water and titrated with 0.025 M NaOH, using a pH meter as indicator. To ensure a good measurement, titration is carried until pH remains stable for at least 10 min. As the acid form is highly hygroscopic, thermogravimetric analysis is used to correct the weight.

(1) $$\mathrm{IEC}=\frac{V_{NaOH}C_{NaOH}}{W_{PDry}}$$

### 2.4. Polymeric Membranes and Conductivity Measurements

Polymeric membranes were fabricated by a solvent casting method, using 2 mL of a 10% polymer aqueous solution on a polystyrene Petri dish and left to dry in a desiccator until membrane could be taken with tweezers; usually 24–36 h are required depending on the degree of protonation. The water content was measured with a TA Instruments TGA-Q500 (TA Instruments, New Castle, DE, USA) using a heat rate of 5 °C/min under nitrogen or air/nitrogen (40/60) atmosphere and membrane thickness was determined with a high precision electronic caliper.

**Figure 1:**
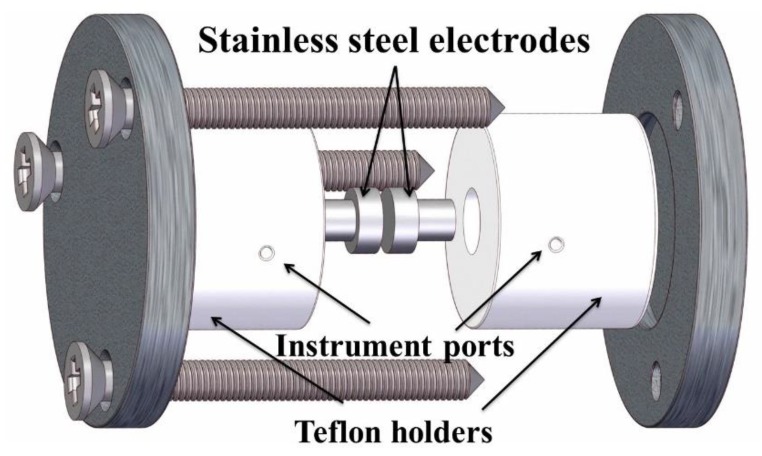
Cylindrical conductivity cell sketch.

Through-plane conductivity ($\sigma_{⊥}$) was measured using a lab made two-electrode conductivity cell (see Figure 1) connected to an AUTOLAB PGSTAT302 equipped with a FRA32M impedance module in the range 1–10 MHz, the resistance value was taken from the fitted spectrum using an equivalent circuit of the type LR (QZ) and $\sigma_{⊥}$ was calculated using Equation (2), where *l* is the membrane thickness, *R* is its resistance and *A* is the electrode area.

(2) $$\sigma_{⊥}=\frac{l}{RA}$$

## 3. Results and Discussions

As the NaSS showed poor solubility in highly concentrated DMF systems at room temperature, but complete solubility when heated to 90 °C, the same heat treatment was used in all cases. Also, because the BPO is poorly soluble in water (9.1 mg/L [23], 0.16 mg/kg [24,25]), the initiator was previously dissolved in 1 mL of DMF and added to the system via syringe once polymerization temperature is reached. No signs of precipitation were observed when adding the initiator (i.e., no turbidity), which indicates a good dissolution of the initiator in the mixture. A pre-polymerization process is performed at 90 °C since bubbles can appear at higher temperatures, caused by the rapid decomposition of the initiator at temperatures above 95 °C and possible boiling of the water. The system is then heated to 105 °C and allowed to polymerize for another 16 h to ensure a high conversion. As shown in Table 1, in the high concentrated DMF systems (i.e., 1/9 and 2/8) a precipitated gel was observed after only ~6 h of polymerization, but in the low concentrated DMF systems (9/1 and 8/2) the gel precipitates after 17 h.

The lower values of precipitation times (*t_p_*), recovery, and molecular weight in the DMF rich systems (Table 1) can be attributed to the lower solubility of the polymer as the chain grows. In contrast, the higher solubility of the polymer in the richer water systems allows the polymer to grow for longer time. As the growing molecules reach a critical solubility limit, the intramolecular interactions become prominent and they begin to precipitate, and since they are partially soluble in the solvent mixture the precipitation takes place in a swollen gel form. This gel precipitation has two main advantages, the easy extraction of the product (avoiding complex post-processing [14,16,26]) and that the obtained polymer is free of residual monomer and initiator since those have a high probability to stay in the solution. Another impurity to consider is the DMF traces, which stay in the gel, interacting with the sulfonic groups [21] and affecting the conductivity [22]. In Table 1, the recovery (%) refers to the dry polymer after the first, second, and third fiber precipitations with acetone. As can be seen after each washing process a loss of product occurs in all cases.

To get rid of the DMF traces, we take advantage of three properties: (1) the ability of the PNaSS to form fibers (a common quality of many polymers), (2) The PNaSS insolubility in acetone (owing by the high sodium salt concentration), and (3) the high miscibility of the DMF with acetone. Therefore, the gel is first dissolved in water and added dropwise to the acetone vortex. Unlike other reports, where the product precipitates as solid particles [16,18,19,20], with this process, polymer fibers are produced and, as stirring continues, they form a woolen-like sponge, easy to extract with tweezers or by decantation. This precipitation is induced by the water presence that helps in the stretching of the chains while the acetone extracts both the water and the DMF. Since the initiator is soluble in acetone and the monomer is not able to form fibers, it is highly probable that residual traces stay in the solvent, so the recovered polymer is less contaminated with these traces. The sponge is finally left in clean acetone to avoid rehydration and afterwards it is dried under vacuum at 50 °C overnight. To ensure the complete removal of contaminants, the recovered sponge is redissolved and reprecipitated two times more.

The obtained results (Table 1) show that the sample PNaSS82 (with W/DMF: 8/2 volume ratio) is the optimum system in terms of highest recovery and molecular weight. At W/DMF ratios higher in DMF than the optimum value the recovery, polymerization time and molecular weight decrease because of the lower solubility of the polymer in DMF [19]. The lower recovery in the case of the sample PNaSS91 compared with that of PNaSS82, can be due to the lower solubility of the initiator in the rich water solvent mixture, which can affect its distribution in the medium.

The obtained dispersities are low compared with those expected for free radical polymerization and especially using BPO as initiator, where DMF plays an important role in the control of the molecular weight. As seen in Table 1, as DMF content increases, the *Ð_M_* decreases, reaching a value as low as 1.15 in the case of the sample PNaSS19, comparable to that of complex procedures of controlled polymerization as living free-radical [28], ATRP [14,17,29], or RAFT [17,18]. These outcomes can be interpreted as “solubility controlled polymerizations”, as far as the biggest molecules stop growing in the solubility limit and permit the smaller molecules to grow for a longer time, and at the same time, the smallest molecules that suffered from rapid termination will stay in solution. Also, a dispersity increase is observed in the samples PNaSS37 and PNaSS28, which can be attributed to a secondary molecular weight distribution, seen as a shoulder in the chromatogram (not shown). This secondary shoulder is not observed for the sample PNaSS19, which can explain the lowest dispersity and the selectivity at low DMF concentrations.

The synthesis for the optimum sample PNaSS82 is scaled up 10 times in order to have enough material for protonation, membrane preparation, and conductivity measurements. Table 2 shows the results of protonation of PNaSS82 samples carried out by the three different methods and the water content and conductivity of the membranes. According to these results, it is very difficult to obtain a complete protonation of the polymer by simply contacting the sample with the two selected acids.

Although both hydrochloric (PSSH82-HCl) and sulfuric (PSSH82-SA) acids were in excess by twofold, the polymer only reaches IEC values of 1.3 and 3.0, respectively, which means only 24% and 55% of the theoretical exchangeable sites (assuming a theoretical IEC = 5.43 meq/g for the mono-sulfonated styrene). This can be due to the high interaction between the polymer chains forming clusters and avoiding the proton exchange. By adding concentrated sulfuric acid to the polymer solution, an IEC of 4.8 meq/g was reached (PSSH82-SAP), which precipitates the polymer as a light brown gel. Even with this procedure, the theoretical IEC was not reached, which can be attributed to the rapid precipitation of the polymer while adding sulfuric acid and to the possible crosslinking of the polymer chains, observed as a color change from white to brown. It’s also observed that this color change is more noticeable when the exchange is carried out without temperature control giving a water insoluble brown product.

This behavior is also observed when the membranes are formed using elevated temperatures, turning them to a black and brittle material, and thus, the membrane preparation must be done inside a desiccator without heating. All samples were prepared under the same conditions, keeping the polymer solution concentration almost the same and using the same time in the desiccator, so the differences in the water content, calculated from TGA curves, are attributable to the differences in the acidic groups content. For example, when the lyophilized PSSH-SAP sample, with the highest acid content, is left outside of the desiccator, it forms a solution with a water content of 75% while the others only absorb a 25%.

To measure the through-plane conductivity ($\sigma_{⊥}$), a specially designed cell was used and coupled to the impedance equipment, which ensures well aligned electrodes with even surfaces. By means of EIS, a resistance of only 15 mΩ was measured for the short-circuited cell, which ensures good measurements of conductivities as high as 2.6 S/cm, almost 30 times higher than that observed for Nafion 117 [30,31,32]. Using this two-electrode configuration, the conductivities of Nafion 117 membranes measured at three different temperatures were in the range of 31.5 to 68.9 mS/cm and used as references.

**Figure 2:**
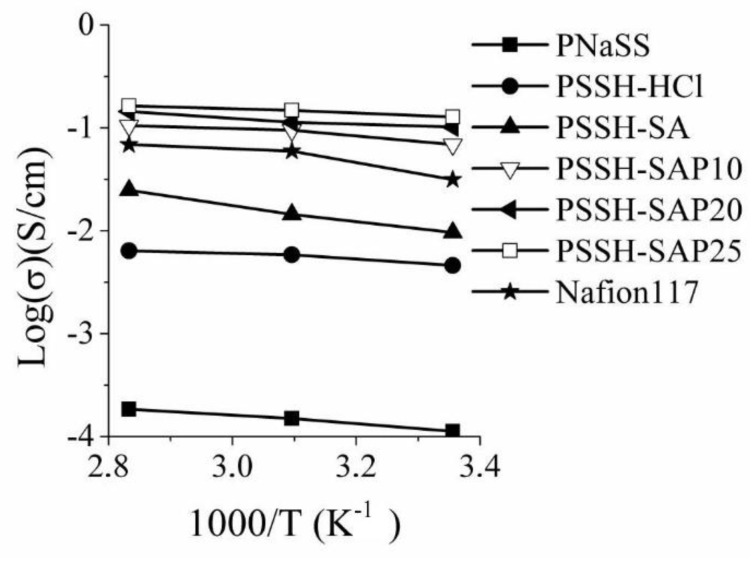
Through-plane conductivity vs temperature for membranes in acid and salt forms.

As observed in Table 1 and Figure 2, the conductivity of the membranes increases as the polymer IEC, the membrane water content, and temperature increase, mostly because the higher concentration and higher mobility of H^+^, compared with that of the Na^+^ [24]. The full salt form of the polymer has the lowest conductivity (0.18 mS/cm at 80 °C) while the PSSH-SAP25, with the highest IEC and water content, has the highest conductivity (164.3 mS/cm at 80 °C), higher than that found for Nafion 117 reference.

## 4. Conclusions

Polymerization of sodium 4-styrenesulfonate in water/DMF solutions, with inexpensive BPO initiator, followed by the fiber precipitation method reported in this paper, was shown to be an easy and efficient option to obtain a clean homopolymer. This method can be interpreted as “solubility controlled” free radical polymerization, as shown by the relatively low dispersities in most cases, with the advantage that high molecular weight can be obtained and atmosphere control is not required. While in the richest DMF system the polymerization has a more controlled behavior, the poorest DMF system is less controlled but more efficient and with higher molecular weight. Even if full protonation of the polymer in solution was not possible, a very high IEC and high conductivities were achieved for the samples precipitated with concentrated sulfuric acid.

## Figures and Tables

**Table 1:** Polymerization data and results.

System	W/DMF	*t_p_*^a^ (h)	Recovery (%) ^b^ (1st; 2nd; 3rd)	M_w_ (kDa) (1st)	*Ð_M_*^c^ (1st)
PNaSS91	9/1	17	93; 90; 88	680	1.85
PNaSS82	8/2	16	98; 96; 95	748	1.85
PNaSS73	7/3	14	96; 94; 90	450	1.52
PNaSS64	6/4	13	96; 93; 90	500	1.54
PNaSS46	4/6	9	92; 90; 84	376	1.40
PNaSS37	3/7	9	85; 83; 80	355	1.74
PNaSS28	2/8	6	70; 72; 78	364	1.66
PNaSS19	1/9	6	60; 58; 53	195	1.15

^a^ Time at which gel precipitation is observed, counting the first 2 h of pre-polymerization. ^b^ Recovery. ^c^ Dispersity [27].

**Table 2:** Through-plane conductivity of the membranes.

Sample	IEC ^a^ meq/g	Water ^b^ (%)	T ^c^ (°C)	*σ*_⊥_ (mS/cm)
PNaSS	0	9	25	0.11
			50	0.15
			80	0.18
PSSH82-HCl	1.3	10	25	4.62
			50	5.85
			80	6.40
PSSH82-SA	3.0	16	25	9.63
			50	14.4
			80	24.7
PSSH82-SAP10	4.8	10	25	68.9
			50	95.2
			80	105.2
PSSH82-SAP22	4.8	22	25	102.4
			50	113.5
			80	144.8
PSSH82-SAP28	4.8	28	25	127.8
			50	148.4
			80	164.3
Nafion117	0.9	6	25	31.5
			50	59.7
			80	68.9

^a^ Measured for the starting polymer; ^b^ Measured for the membranes; ^c^ Temperature for $\sigma_{⊥}$ measurement.
